# Prenatal exposure to maternal smoking and adult lung cancer risk: a nested case-control study using peripheral blood leukocyte DNA methylation prediction of exposure

**DOI:** 10.1093/eep/dvae015

**Published:** 2024-09-20

**Authors:** Meng Ru, Dominique S Michaud, Naisi Zhao, Karl T Kelsey, Devin C Koestler, Jiayun Lu, Elizabeth A Platz, Christine M Ladd-Acosta

**Affiliations:** Department of Epidemiology, Johns Hopkins Bloomberg School of Public Health, Baltimore, MD 21205, United States; School of Medicine, Tufts University, Boston, MA 02111, United States; School of Medicine, Tufts University, Boston, MA 02111, United States; Departments of Epidemiology and Pathology and Laboratory Medicine, Brown University, Providence, RI 02912, United States; Department of Biostatistics & Data Science, University of Kansas Medical Center, Kansas City, KS 66160, United States; Department of Epidemiology, Johns Hopkins Bloomberg School of Public Health, Baltimore, MD 21205, United States; Department of Epidemiology, Johns Hopkins Bloomberg School of Public Health, Baltimore, MD 21205, United States; Department of Epidemiology, Johns Hopkins Bloomberg School of Public Health, Baltimore, MD 21205, United States

**Keywords:** prenatal smoking, DNA methylation, lung cancer

## Abstract

A prior study reported no association between prenatal smoking methylation scores and adult lung cancer risk adjusting for methylation-predicted adult smoking, without considering maternal smoking trends by birth cohort. To address this gap, we examined the association between prenatal smoking methylation scores and adult lung cancer, independent of methylation-predicted adult packyears and by birth cohort, in a study nested in CLUE II. Included were 208 incident lung cancer cases ascertained by cancer registry linkage and 208 controls matched on age, sex, and smoking. DNA methylation was measured in prediagnostic blood. We calculated two prenatal smoking scores, using 19 (Score-19) and 15 (Score-15) previously identified CpGs and a methylation-predicted adult packyears score. Conditional logistic regression was used to estimate odds ratios (ORs) and 95% confidence intervals (CIs) adjusting for adult packyears score and batch effects. Score-15 was positively associated with lung cancer (per standard deviation, OR = 1.40, 95% CI = 1.10–1.79, *P*-trend = .006), especially in the 1930–1938 birth cohort (OR = 3.43, 95% CI = 1.55–7.60, *P*-trend = .002). Score-19 was associated only in the 1930–1938 birth cohort (OR = 2.12, 95% CI = 1.15–3.91). Participants with both prenatal scores below the median (vs all other combinations) had lower risk (OR = 0.44, 95% CI = 0.27–0.72), especially in the 1930–1938 birth cohort (OR = 0.16, 95% CI = 0.04–0.62). Among ever smokers, participants with higher prenatal smoking scores had higher risk, irrespective of adult packyears (low: OR = 2.81, 95% CI = 1.38–5.72, high: OR = 2.67, 95% CI = 1.03–6.95). This prospective study suggests a positive association between prenatal smoking exposure and adult lung cancer risk, especially in the 1930–1938 birth cohort, independent of active smoking. Future studies with multiple birth cohorts are needed.

## Introduction

The prenatal period is a critical window for development, and exposures during this period may influence health and diseases across the life course, including cancer [1, 2]. In 2017, ∼7% of women in the USA smoked during pregnancy with higher prevalence in the past [3–5]. Because active smoking alone does not explain all lung cancer cases [6] and not all smokers develop lung cancer [7], it is possible that prenatal smoking exposure may contribute to adult lung cancer risk independent of adult active smoking and possibly act synergistically with active smoking.

Prenatal smoking may increase the risk of cancer development in adulthood through the (pro)toxicants present in cigarettes that can cross the placental barrier and cause potential genotoxic effects on fetal cells [8–10]. Animal models [11] and human studies [12] suggest that prenatal exposure to smoking increases the offspring’s su7hood,including common cancers such as colon and breast cancers. Also, prenatal smoking could be indirectly linked to an increased adult cancer risk through the adverse effects on decreased birth weight [13], i.e. low birth weight may mediate prenatal exposure effects on later incident cancer risk. Evidence supporting this includes registry recorded low birth weight association with increased risk of ovarian cancer in adults [14]. It should be noted, however, that some studies did not find lower self-reported birth weight significantly associated with increased risk of other cancers, especially breast cancer [15, 16]. While generally supportive of our hypothesis that prenatal exposure to smoking increases risk of adult-onset lung cancer, the previous epidemiologic studies were limited by small sample sizes or insufficient follow-up time for cancer incidence, lack of prospective collection of prenatal exposure, inability to assess internal biologic dosimeters of exposure, or studying only childhood cancers [17–24].

Direct measurement of prenatal smoking exposure in prospective cohort studies that recruit adults remains a considerable challenge. Real-time data collection across the life course starting in the prenatal period is typically not feasible in large-scale prospective studies, and collection of prenatal exposure in adults is susceptible to substantial exposure misclassification or recall bias. As a possible solution, recent studies have identified specific CpG sites where DNA methylation levels were associated with prenatal smoking [25–31]. These studies reported DNA methylation signatures of prenatal smoking exposure that persist into childhood [25–29] and even into adulthood [30, 31], potentially allowing for investigations of prenatal smoking exposure and risk of adult diseases in epidemiologic studies that begin in adulthood in a reliable way. Richmond *et al*. [30] showed that a methylation score built using a subset of 19 CpGs identified in neonates [26] (which we refer to as Score-19) related to their prenatal smoking exposure that was also significantly different in samples collected at mid-childhood could also be detected in blood collected from an independent set of participants enrolled in the Avon Longitudinal Study of Children and Parents (ALSPAC), at 30–54 years of age. They further showed that this signature was independent of personal active smoking history, paternal prenatal, and other postnatal secondary smoking exposures [30]. Additionally, they showed that a separate highly accurate methylation score for predicting personal smoking history was not able to predict prenatal smoking exposure with high accuracy (Area under the curve = 0.57) [30], suggesting that the prenatal smoking exposure methylation signatures are specific to that critical window and mode of exposure. Because Score-19 was developed in neonate cord blood, and many studies are interested in having an adult peripheral blood marker of prenatal smoking, Richmond *et al*. also identified a new signature of prenatal smoke exposure in peripheral blood biospecimens collected from adults at 30 years of age or older; 15 CpGs were identified [30] (which we refer to as Score-15) including eight that overlap with those from neonates. These overlapping CpGs were in *MYO1G* (four CpGs), *CNTNAP2* (one CpG), *CYP1A1* (one CpG), *FRMD4A* (one CpG), and *TIFAB* (one CpG) genes. Thus, evidence supports that methylation biomarkers can reflect prenatal smoking exposure and may provide a feasible surrogate tool to evaluate the effect of prenatal smoking exposure on health outcomes using existing prospective cohorts with blood leukocyte methylation data collected outside of the prenatal time window [32].

A recent nested case-control study investigated the associations between prenatal smoking methylation signatures and adult cancer risk in Australian and European populations. The authors reported null or inverse associations with lung cancer adjusting for methylation-predicted adult active smoking [33]. However, that study did not consider the notable secular trends in maternal smoking prevalence by birth cohort before the 1960s in the USA, UK, and similar higher-income countries [4]. Smoking prevalence increased from few women smoking at the turn of last century to 25–40% from World War II to the late 1950s in the USA [4]. To our knowledge, information on the national prevalence of smoking during pregnancy is not available during that timeframe. It is thus likely that people born before the 1960s might have had a changing opportunity for exposures to prenatal smoking due to the historical variability of the prevalence of women smoking. No studies have evaluated this effect in the US population.

To address gaps in our knowledge and to address previous study limitations when testing the association between prenatal smoke exposure and adult lung cancer risk, we conducted a nested case-control study among participants in the CLUE II cohort from the USA [34–36]. CLUE II included participants born from 1905 to 1961, which allowed us to consider potential birth cohort effects when evaluating the association between methylation scores for prenatal smoking exposure and adult lung cancer risk, independent of methylation-predicted adult packyears. The CLUE II study is a prospective cohort with rigorous ascertainment of incident lung cancer by cancer registry linkage, collection of adult smoking history, and other potential covariates of interest, for which we previously performed leukocyte DNA methylation profiling. We hypothesized that prenatal smoking exposure is associated with increased adult lung cancer risk, independent of adult active smoking, especially in birth cohorts when maternal smoking was more likely to be prevalent due to secular historical trends. We also assessed whether the effects of prenatal smoking exposure differed by personal history of active smoking, specifically hypothesizing that individuals with both prenatal exposure and a high personal history of active smoking would have the greatest lung cancer risk.

## Results

Of the 208 matched case-control pairs, 54% were female and 89% were ever smokers. The median follow-up since CLUE II blood draw was 26 years (range: 0–29 years). Among ever smokers, lung cancer cases had higher self-reported packyears and methylation-predicted adult active smoking score (in packyears smoked) than controls (Supplementary Table S1).

### Distribution of prenatal smoking scores by case and control status and by birth cohort quartiles

We observed higher median prenatal smoking methylation scores among individuals with lung cancer compared to controls (Table 1); this difference was present only in the 1930–1938 birth cohort (Q2, the second most recent birth cohort in the analytic population), and for Score-15, the methylation signature developed in ALSPAC adult biospecimens to reflect prior prenatal exposure [30]. In contrast, the score derived from methylation changes detected in newborns and shown to persist into mid-childhood and early adulthood (Score-19) did not differ between cases and controls overall or by birth cohort.

**Table 1.** Median (Q1, Q3) prenatal smoking scores in 208 pairs of lung cancer cases and matched controls, overall and by birth cohort, nested in CLUE II, 1989

Prenatal smoking score	Case, *N* = 208	Control, *N* = 208	*P* ^*^
Score-15^a^	0.29 (0.27, 0.30)	0.28 (0.26, 0.29)	.01
Birth cohort quartile^b^			
Q1: 1938–1961	0.29 (0.28, 0.30)	0.29 (0.27, 0.30)	.1
Q2: 1930–1938	0.29 (0.27, 0.29)	0.27 (0.25, 0.29)	.02
Q3: 1923–1930	0.27 (0.26, 0.29)	0.28 (0.27, 0.28)	.4
Q4: 1905–1923	0.28 (0.25, 0.29)	0.27 (0.25, 0.29)	1.0
Score-19^a^	0.22 (0.21, 0.24)	0.22 (0.21, 0.23)	.2
Birth cohort quartile^b^			
Q1: 1938–1961	0.23 (0.22, 0.24)	0.23 (0.21, 0.25)	.7
Q2: 1930–1938	0.22 (0.21, 0.24)	0.22 (0.20, 0.23)	.2
Q3: 1923–1930	0.22 (0.20, 0.23)	0.21 (0.21, 0.22)	.3
Q4: 1905–1923	0.22 (0.19, 0.23)	0.21 (0.19, 0.23)	1.0

a Score 15 was generated from CpGs discovered in adult biospecimens that were associated with their prenatal smoking exposure status and shown to be independent of own active personal smoking history or passive secondary sources of smoking exposure [30]. Score-19 uses CpGs that were discovered in neonate cord blood and shown to also be detectable into adulthood and independent of own personal active smoking history [26].

b Quartile (Q) cut points based on the case’s birth year. Matched control was placed in the same quartile as the case.

* *P*-value computed using the Wilcoxon sign rank test.

**Figure 1.**
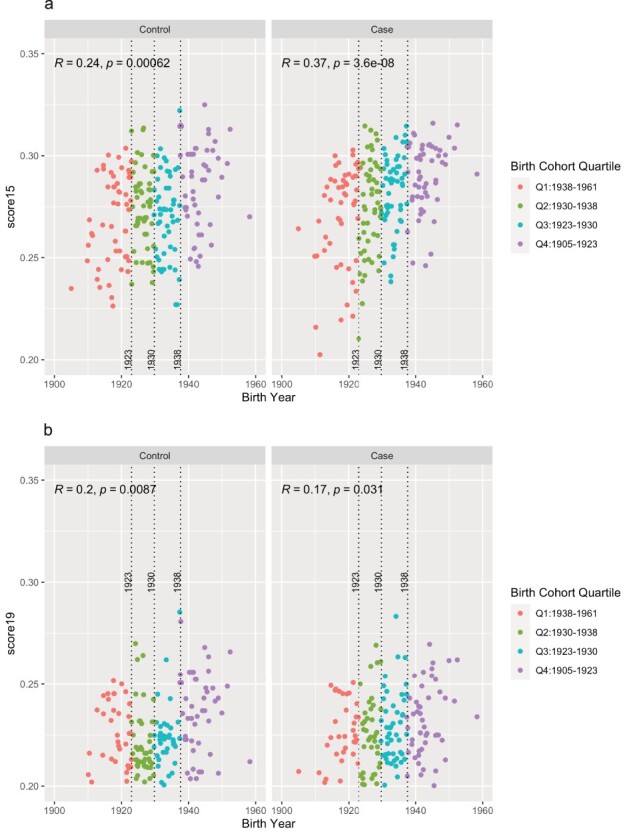
Spearman correlation coefficients (*R*) and *P*-values for the correlation between methylation-based smoking scores, Score-15 and Score-19, and birth year in 208 matched pairs of lung cancer cases and controls, nested in CLUE II, 1989.

Given the historical variability in smoking prevalence in women and the different distributions of methylation smoking scores between cases and controls in the 1930–1938 birth cohort group, we visualized the secular trend in the prenatal smoking scores. As shown in Fig. 1a and b, participants from more recent birth cohorts tended to have higher prenatal smoking scores in both cases and controls. The correlations between the prenatal smoking scores and birth year were modest (*r*: 0.23–0.37) in both the cases and controls, although slightly stronger in the cases. There was no apparent secular trend in the methylation-predicted score of active smoking history (in packyears) by birth cohort in cases and controls (Supplementary Fig. S1).

### Association between prenatal smoking scores and lung cancer risk

Both prenatal smoking methylation scores (Score-15 and Score-19) were positively associated with lung cancer risk [Table 2, per standard deviation (SD), Score-15: odds ratio (OR) = 1.40, 95% confidence interval (CI) = 1.10–1.79; Score-19: OR = 1.22, 95% CI = 0.96–1.55]. When stratified by birth cohort group, both scores showed a stronger positive and statistically significant association only in the 1930–1938 birth cohort (per SD, Score-15: OR = 3.43, 95% CI = 1.55–7.60; Score-19: OR = 2.12, 95% CI = 1.15–3.91). When stratified by quartiles of age at diagnosis (rather than by birth cohort), both scores appeared to be positively associated with lung cancer risk in all but the third quartile group of age at diagnosis (i.e. next to oldest age in the diagnosis group). Given that cases who were the same age at diagnosis could be from different birth cohorts, we assessed whether the associations we observed were driven by birth cohort vs age at diagnosis. To do so, we stratified by the joint classification of participants by birth cohort and age at diagnosis. Both prenatal smoking scores appeared to be associated with a higher risk of lung cancer within joint categories that included the 1930–1938 birth cohort (Q2) and either younger or older age at lung cancer diagnosis. However, we also observed that in the joint category that included the earlier birth cohorts (Q4, Q3) and younger ages at lung cancer diagnosis, both prenatal scores appeared to be associated with higher lung cancer risk.

**Table 2.** Associations between prenatal smoking scores and lung cancer risk overall, by birth cohort, and by age at diagnosis, 208 matched pairs nested in the CLUE II cohort (1989–2018).

Score-15^a^					Score-19^a^				
	Number of pairs, *N*	OR^b^	95% CI^b^	*P*		Number of pairs, *N*	OR^b^	95% CI^b^	*P*
Overall	208	**1.40**	**1.10, 1.79**	**.006**	Overall	208	1.22	0.96, 1.55	.1
By birth cohort quartile^c^									
Q1: 1938–1961	52	1.11	0.55, 2.23	.8	Q1: 1938–1961	52	0.74	0.38, 1.43	.4
Q2: 1930–1938	52	**3.43**	**1.55, 7.60**	**.002**	Q2: 1930–1938	52	**2.12**	**1.15, 3.91**	**.02**
Q3: 1923–1930	52	1.07	0.58, 1.99	.8	Q3: 1923–1930	52	1.10	0.64, 1.88	.7
Q4: 1905–1923	52	1.10	0.69, 1.74	.7	Q4: 1905–1923	52	1.05	0.63, 1.74	.9
By age at diagnosis quartile (years)^c^									
Q1: ≤67.2	52	**2.08**	**1.04, 4.17**	**.04**	Q1: ≤67.2	52	1.32	0.75, 2.32	.3
Q2: ≤74.0	52	1.81	0.96, 3.41	.07	Q2: ≤74.0	52	1.30	0.74, 2.29	.4
Q3: ≤79.8	52	0.93	0.56, 1.55	.8	Q3: ≤79.8	52	0.94	0.50, 1.78	.8
Q4: >79.8	52	1.35	0.79, 2.32	.3	Q4: >79.8	52	1.28	0.79, 2.06	.3
By joint categories of birth cohort and age at diagnosis (Dx) quartile (years)^c^									
Birth cohort Q1 and age at Dx Q1–2	48	0.87	0.39, 1.93	.7	Birth cohort Q1 and age at Dx Q1–2	48	0.64	0.30, 1.38	.3
Birth cohort Q2 and age at Dx Q1–2	28	**3.62**	**1.20, 10.90**	**.02**	Birth cohort Q2 and age at Dx Q1–2	28	2.26	0.98, 5.21	.06
Birth cohort Q3–4 and age at Dx Q1–2	28	3.50	0.96, 12.78	.06	Birth cohort Q3–4 and age at Dx Q1–2	28	1.82	0.65, 5.10	.3
Birth cohort Q1–2 and age at Dx Q3–4	28	3.02	0.83, 10.96	.09	Birth cohort Q1–2 and age at Dx Q3–4	28	1.65	0.56, 4.85	.4
Birth cohort Q3–4 and age at Dx Q3–4	76	0.99	0.66, 1.48	1.0	Birth cohort Q3–4 and age at Dx Q3–4	76	1.07	0.71, 1.61	.7

a Score-15 was generated from CpGs discovered in adult biospecimens that were associated with their prenatal smoking exposure status and shown to be independent of own active personal smoking history or passive secondary sources of smoking exposure [30]. Score-19 uses CpGs that were discovered in neonate cord blood and shown to also be detectable into adulthood and independent of own personal active smoking history [26].

b OR: OR per SD. Models were adjusted for BMI, adult packyears score, and PCs for batch effects.

c Matched control was placed in the same quartile as the case.

Bold values denote statistical significance at the *P* < 0.05 level

Among ever smokers (89% of pairs), the positive association between both scores and risk of lung cancer persisted in the 1930–1938 birth cohort (Table 3, per SD, Score-15: OR = 2.78, 95% CI = 1.30–5.96; Score-19: OR = 1.86, 95% CI = 1.00–3.46). When restricting to non–small cell lung cancer (NSCLC) cases and matched controls (75% of pairs), the pattern of the association for the scores was similar by birth cohort quartile to total lung cancer cases analysis. In the sensitivity analysis excluding cases diagnosed within 5 years after the blood draw, the results were similar, with the overall positive association modestly attenuated (but for Score-15, it remained statistically significant), but the association in the 1930–1938 birth cohort appeared to strengthen (Supplementary Table S2).

**Table 3.** Associations between prenatal smoking scores and lung cancer risk overall and by birth cohort, among ever smokers (186 pairs) and for NSCLC (155 pairs), nested in the CLUE II study (1989–2018).

Score-15^a^									Score-19^a^								
	Ever smokers				NSCLC					Ever smokers				NSCLC			
	Number of pairs, *N*	OR^b^	95% CI^b^	*P*	Number of pairs, *N*	OR^b^	95% CI^b^	*P*		Number of pairs, *N*	OR^b^	95% CI^b^	*P*	Number of pairs, *N*	OR^b^	95% CI^b^	*P*
Overall	186	1.40	1.08, 1.81	.010	155	1.13	0.84, 1.50	.4	Overall	186	1.21	0.93, 1.56	.2	155	0.92	0.69, 1.23	.6
Q1: 1938–1961	49	0.95	0.45, 2.00	.9	43	1.04	0.49, 2.18	1.0	Q1: 1938–1961	49	0.57	0.27, 1.22	.2	43	0.73	0.38, 1.43	.4
Q2: 1930–1938	46	2.78	1.30, 5.96	.008	40	2.46	1.07, 5.63	.03	Q2: 1930–1938	46	1.86	1.00, 3.46	.05	40	1.62	0.80, 3.29	.2
Q3: 1923–1930	46	1.01	0.53, 1.95	1.0	34	1.19	0.57, 2.46	.6	Q3: 1923–1930	46	1.08	0.62, 1.90	.8	34	1.09	0.58, 2.02	.8
Q4: 1905–1923	45	1.31	0.77, 2.24	.3	38	0.73	0.38, 1.41	.4	Q4: 1905–1923	45	1.18	0.63, 2.19	.6	38	0.52	0.24, 1.14	.10

a Score-15 was generated from CpGs discovered in adult biospecimens that were associated with their prenatal smoking exposure status and shown to be independent of own active personal smoking history or passive secondary sources of smoking exposure [30]. Score-19 uses CpGs that were discovered in neonate cord blood and shown to also be detectable into adulthood and independent of own personal active smoking history [26].

b OR: OR per SD. Models were adjusted for BMI, adult packyears score, and PCs for batch effects.

When assessing the joint categories of the two prenatal smoking scores irrespective of the birth cohort, participants with one (low/high) or both scores (high–high) above the median had a significantly and similarly higher risk of lung cancer, compared with having both scores below the median (low–low, Table 4). These associations were strongest in the 1930–1938 birth cohort (Table 4). Given that the low/high and high–high groups had similar associations, we estimated the association for the low–low group compared with the other groups combined. The OR was 0.44 (95% CI = 0.27–0.72) combining all birth cohorts, and in each birth cohort, the OR for this association was inverse but was statistically significant only in the 1930–1938 birth cohort (OR = 0.16, 95% CI = 0.04–0.62).

**Table 4.** Associations between joint categories of two prenatal smoking scores, Score-15 and Score-19^a^, and lung cancer risk overall and by birth cohort, 208 matched pairs nested in the CLUE II cohort (1989–2018).

	Cases/controls	OR (95% CI)^b^	*P*	OR (95% CI)^b^ (non-low–low^a^ as reference)	OR (95% CI)^b^ (low/high^a^ as reference)
Overall					
Low–low^a^	72/173	Reference		0.44 (0.27–0.72)	0.43 (0.23–0.81)
Low/high^a^	40/72	2.32 (1.24–4.34)	0.008	Reference	Reference
High–high^a^	96/171	2.23 (1.31–3.78)	0.003		0.96 (0.52–1.78)
Birth cohort quartile: 1938–1961					
Low–low	9/24	Reference		0.70 (0.21–2.33)	0.59 (0.13–2.73)
Low/high	8/17	1.70 (0.37–7.86)	0.5	Reference	Reference
High–high	35/63	1.31 (0.36–4.73)	0.7		0.77 (0.19–3.19)
Birth cohort quartile: 1930–1938					
Low–low	18/45	Reference		0.16 (0.04–0.62)	0.17 (0.03–0.80)
Low/high	11/21	6.05 (1.25–29.2)	0.03	Reference	Reference
High–high	23/38	6.12 (1.46–25.6)	0.01		1.01 (0.26–3.97)
Birth cohort quartile: 1923–1930					
Low–low	22/54	Reference		0.32 (0.09–1.13)	0.49 (0.10–2.32)
Low/high	10/16	2.04 (0.43–9.67)	0.4	Reference	Reference
High–high	20/34	4.67 (0.96–22.8)	0.06		2.29 (0.38–13.7)
Birth cohort quartile: 1905–1923					
Low–low	23/50	Reference		0.57 (0.22–1.52)	0.25 (0.06–1.00)
Low/high	11/18	3.98 (1.00–15.9)	0.050	Reference	Reference
High–high	18/36	0.99 (0.31–3.15)	1.0		0.25 (0.05–1.15)

a Score-15 was generated from CpGs discovered in adult biospecimens that were associated with their prenatal smoking exposure status and shown to be independent of own active personal smoking history or passive secondary sources of smoking exposure [30]. Score-19 uses CpGs that were discovered in neonate cord blood and shown to also be detectable into adulthood and independent of own personal active smoking history [26]. Low–low: Both Score-15 and Score-19 below the median. Low/high: One of the Score-15 and Score-19 below the median, the other greater or equal to the median. High–high: Both Score-15 and Score-19 greater or equal to the median.

b OR: OR per SD. Models were adjusted for BMI, adult packyears score, and PCs for batch effects.

### Joint association of prenatal smoking scores and adult packyears scores with lung cancer risk among ever smokers

We performed this analysis restricting to ever smokers: participants with higher prenatal smoking scores (low/high or high–high as defined in Table 4) had a higher lung cancer risk, irrespective of their adult smoking levels (higher or lower in the methylation-predicted adult active smoking scores in packyears smoked) compared with having lower prenatal smoking scores (low–low as defined in Table 4) and lower adult packyears scores (Supplementary Table S3).

To better understand the influence of the individual CpGs comprising the two prenatal smoking scores on lung cancer risk, we decomposed the prenatal smoking scores to consist of the genes that have also been found to be associated with current active smoking status: *AHRR, MYO1G, CYP1A1, FRMD4A*, and *GFI1*. For each gene we built (I) a gene-only methylation score that consisted of the subset of CpG sites in that gene from the overall prenatal smoking scores and (II) a no-gene methylation score that excluded all CpG sites located in that specific gene under investigation. We observed that the *AHRR*-only score was positively associated with lung cancer risk (Table 5, 2 CpGs, per SD, OR = 1.80, 95% CI = 1.18–2.74). Score-19 excluding *AHRR* CpGs yielded a similar OR (16 of 18 CpGs available, OR = 1.19, 95% CI = 0.92–1.53). Similarly, we created scores for the four CpGs in *MYO1G*, five CpGs in *CYP1A1*, three CpGs in *FRMD4A*, and one CpG in *GF1*. *MYO1G*-only Score-15 was positively associated with lung cancer risk (three CpGs available, OR per SD = 1.42, 95% CI = 1.09–1.85). After excluding *MYO1G* CpGs from Score-15, the positive association with lung cancer risk (10 of 13 CpGs available, OR = 1.15, 95% CI = 0.93–1.44) was attenuated relative to the association for the original Score-15. The same pattern was observed for *MYO1G* in Score-19. Excluding *CYP1A1, FRMD4A* and *GF1* CpGs did not affect the association of Score-15 and Score-19 with lung cancer risk, and only the *FRMD4A* CpG score was associated with lung cancer risk (Table 5).

**Table 5.** Association between gene-level methylation scores (AHRR*, MYO1G, CYP1A1, FRMD4A, and GFI1*) and lung cancer risk, among ever smokers (186 matched pairs) nested in the CLUE II cohort (1989–2018).

Score-15^a^				Score-19^a^			
	OR^b^	95% CI^b^	*P*-value		OR^b^	95% CI^b^	*P*-value
Original Score-15: per SD	1.40	1.08, 1.81	.01	Original Score-19: per SD	1.21	0.93–1.56	.2
				*AHRR*-only*	1.80	1.18–2.74	.006
				Score-19, no *AHRR*	1.19	0.92–1.53	.2
*MYO1G*-only	1.42	1.09, 1.85	.01	*MYO1G*-only	1.38	1.06, 1.80	.02
Score-15, no *MYO1G*	1.15	0.93, 1.44	.2	Score-19, no *MYO1G*	0.99	0.77, 1.27	.9
*CYP1A1*-only	0.97	0.77, 1.23	.82	*CYP1A1*-only	0.98	0.77, 1.25	.86
Score-15, no *CYP1A1*	1.41	1.09, 1.82	.01	Score-19, no *CYP1A1*	1.31	1.01, 1.70	.04
*FRMD4A*-only	1.21	0.96, 1.52	.1	*FRMD4A*-only	1.13	0.90, 1.41	.3
Score-15, no *FRMD4A*	1.39	1.09, 1.81	.01	Score-19, no *FRMD4*	1.19	0.92, 1.53	.2
				*GFI1*-only*	0.92	0.72, 1.16	.5
				Score-19, no *GFI1*	1.22	0.95, 1.58	.1

*Notes*: These genes were selected for their previously found association with active smoking related.

* In Score-19: *AHRR* and *GFI* CpGs were only included in Score-19. Only one CpG on *AHRR* was included in Score-15 but not available in our data. In Score-19, two CpGs available were on *AHRR*, three available on *MYO1G*, five from *CYP1A1*, three from *FRMD4A*, and one from *GFI*. In Score-15, three CpGs available were on *MYO1G*, one on *CYP1A1*, and one on *FRMD4A*.

a Score-15 was generated from CpGs discovered in adult biospecimens that were associated with their prenatal smoking exposure status and shown to be independent of own active personal smoking history or passive secondary sources of smoking exposure [30]. Score-19 uses CpGs that were discovered in neonate cord blood and shown to also be detectable into adulthood and independent of own personal active smoking history [26].

b OR: OR per SD. Models were adjusted for BMI, adult packyears score, and PCs for batch effects.

## Discussion

In this prospective, nested case-control study, higher peripheral blood leukocyte DNA methylation–predicted prenatal smoking scores were associated with a higher risk of lung cancer in adulthood. This association was consistently strong in the 1930–1938 birth cohort, even when adjusting for methylation-predicted active smoking history (in packyears smoked) and when restricting to ever smokers and NSCLC. Our findings appear to support the hypothesis that prenatal smoking may increase the risk of lung cancer in adulthood although confirmation is needed.

Multiple studies have developed signatures of prenatal smoking based on DNA methylation alterations [25–31]; however, only one other study has used these signatures to investigate the associations with future cancer risk in adulthood [33]. A nested case-control study of multiple cancer sites, observed OR < 1 for the associations between five methylation prenatal smoking scores (including Score-15 and Score-19 that we also used) and lung cancer risk; the inverse association was statistically significant only after adjusting for adult methylation-predicted active smoking history (in packyears). The study conducted in Australia included participants mostly born between 1920 and 1955. Our study, which observed a positive association, included participants in birth cohorts that overlapped with that study and was conducted in one US state. While we are unsure of why the associations in the two studies are in opposite directions, differences between the studies could have contributed to the contrasting results, such as possibly different maternal smoking prevalence in the two countries; while authoritative information is not available for those birth cohorts, the peak smoking prevalence of women born in the 1930s was lower and occurred later in Australia (about 24% in the late 1960s) compared to that in the USA (about 35% in the mid-1950s) based on an Australian report [37]. Additionally, different methylation sequencing platforms were used in the two studies, which resulted in different numbers of CpG sites included in the scores. Comparing whether the distributions of the scores in our study are similar to those in the Australian study would help inform both these possibilities, but they reported scores rescaled to *Z*-scores [33], while we reported the original scores, thereby making it impossible to compare. Chance differences also remain a possibility.

Due to varying smoking prevalences among women across the early to mid-20th century, people in different birth cohorts may have different likelihoods of exposures to prenatal smoking, which could attenuate the associations. Thus, we stratified the analyses by birth cohort. While we cannot rule out the role of chance for a subgroup finding, we found that the association was consistently strong in one of the birth cohorts, which overlapped in time with the Great Depression in the USA. It is possible that the effect of maternal smoking on adult lung cancer risk in the 1930–1938 birth cohort was exacerbated by maternal malnutrition or other era-specific factors experienced by the fetus. For example, biomass burning was prevalent as the primary heating method in some eras in the USA (around 75% in the 1940s [38]). Biomass smoke shares many constituents with tobacco smoke. We are not able to determine the extent to which prenatal smoking scores may also capture other such exposures. Future studies should consider stratifying by birth cohort, potentially exploring different cutoffs of birth cohort based on the changing prevalence of smoking by women over calendar time, by historical events and influence of other exposures, such as biomass burning, on the prenatal smoking scores.

We also observed that the association between the prenatal score and lung cancer risk appeared to differ among strata of age at diagnosis. Because the distribution of age at diagnosis is constrained by range of birth cohorts included in the analysis, we examined the prenatal score association by joint categories of age in the lung cancer diagnosis birth cohort to assess which is the more important effect modifier. The findings tended to support the fact that birth cohort was the more important effect modifier given that prenatal smoking score association with lung cancer was present for the joint categories that included the 1930–1938 birth cohort irrespective of age at diagnosis. We also did observe a positive association for the score in the joint category that included earlier birth cohorts (Q4, Q3) and younger ages at lung cancer diagnosis.

To increase the specificity of classifying prenatal smoking exposure and to address the potential limitation of narrow ranges of the prenatal smoking scores leading to null findings, we created joint categories from both prenatal smoking scores using the median as the cutpoint. We hypothesized that the joint categories would better differentiate the exposed and the unexposed (i.e. participants with both scores under the median (“low–low group”) would be less likely to have experienced prenatal smoke exposure). We found that participants in the low–low group had significantly lower lung cancer risk compared to those in all other groups combined, especially in the 1930–1938 birth cohort. We also assessed the combined effects of prenatal smoking and adult active smoking on lung cancer risk using the joint categories of prenatal smoking (using both scores: low–low group vs others) and adult methylation-predicted active smoking history (in packyears, cut point: median or 67th percentile). Restricted to ever smokers only (not stratified by birth cohort due to sample size), a higher prenatal smoking score was associated with higher lung cancer risk, irrespective of the levels of active smoking history in methylation-predicted packyears, implying that prenatal smoking exposure might add risk to adult active smoking on lung cancer risk.

The prenatal smoking scores used in this study were developed to mark exposure, irrespective of their associations with future cancer risk or other health outcomes. To further determine the role of certain CpGs associated with both active smoking and prenatal smoking in the association with adult lung cancer risk [39–41], we decomposed the prenatal smoking scores by gene (*AHRR*, *MYO1G*, and *CYP1A1*) and estimated their associations with lung cancer risk among ever smokers. While the association for the *AHRR* CpG (cg05549655) from the prenatal smoking score was strongly associated with adult lung cancer risk, excluding it from the prenatal smoking score did not eliminate the positive association for Score-19, suggesting that prenatal smoking rather than active smoking remains the explanation for the positive association between Score-19 and lung cancer risk. Excluding the CpGs in *MYO1G* eliminated the positive association of Score-15 and Score-19 with lung cancer risk, suggesting that prenatal-associated CpGs on *MYO1G* might have a notable association with lung cancer risk. Since *MYO1G* is a member of the motor protein—class I myosin family and abundant in T and B lymphocytes and mast cells—it might indicate that prenatal smoking exposure might affect the mobility of leukocytes [42–45] in the offspring’s lung cancer development. Future studies are needed to further investigate this hypothesis.

This study has many strengths including the prospective design, the leveraging of existing DNA methylation data, and persistent, prenatal specific, methylation signature as a surrogate for unmeasured history of maternal smoking during gestation and well-documented lung cancer cases. We were able (i) to conduct analyses stratified by birth cohort to account for the secular trends in the prevalence of smoking among women in the early to mid-20th century, (ii) to increase the specificity of classification of prenatal smoke exposure by using two scores jointly, and (iii) to assess the influence of prenatal smoke exposure separate from the effects of active smoking.

This study has several possible limitations as well. First, because our methylation data were profiled using a different platform (850K MethylationEPIC) than the one used in the development of the methylation signatures (HM450), some CpG sites were missing from Score-15 (*n* = 1) and Score-19 (*n* = 2), due to platform coverage differences. Thus, these modified scores may have some nondifferential measurement error, which may have attenuated the association between these scores and lung cancer risk. Second, we were not able to assess the influence of maternal smoking before and after pregnancy; paternal smoking before, during, and after the mother’s pregnancy; or the offspring’s smoking during adolescence on Score-15 and Score-19 or on the associations we observed. However, previous studies have shown evidence supporting the specificity of these methylation signatures to maternal smoking during pregnancy. In the development of Score-15, the investigators showed that the associations between maternal smoking and the selected CpGs were not attenuated after adjusting for paternal smoking (smoker/nonsmoker) and that the association between maternal smoking and the selected CpGs was substantially stronger than the association between paternal smoking and the same CpGs [30]. Also, in the development of Score-19, the investigators included CpGs that persisted into young childhood, which predated the potential initiation of the offspring’s smoking in adolescence [26]. Third, we could not assess other exposures related to lung cancer, such as radon, organic burning materials other than tobacco, or geographic/context-specific factors as confounders. At this time, whether these exposures correlate with the prenatal smoking scores has not been investigated to our knowledge. Fourth, our lung cancer nested case-control study was of modest size, and thus chance associations and null findings cannot be ruled out. Finally, we cannot determine the generalizability of this study because it was conducted in a single geographic location, where the majority of residents were White.

## Conclusion

This prospective study suggests a positive association between prenatal smoking exposure predicted by leukocyte DNA methylation scores and adult lung cancer risk, especially in the 1930–1938 birth cohort. This study may inform lung cancer risk variability among ever active smokers. Also, this study supports the feasibility of studying prenatal exposures in relation to risk of adult-onset diseases using DNA methylation signatures in peripheral blood leukocytes, which is critical for cohort studies in which information on *in utero* exposures are generally not available. Large consortial studies that include multiple birth cohorts, and in locations or populations with differing or emerging smoking prevalence among pregnant people, and that can take into account active smoking will be needed to confirm this association. In addition, studies are needed to evaluate the influence of maternal and paternal smoking before, during, and after pregnancy with that child and the child’s own active smoking in later childhood and adolescence.

## Materials and methods

### Study population and design

This study was nested in the CLUE II cohort among those who also participated in the CLUE I cohort and did not have a prior cancer diagnosis at the time that they were recruited into CLUE II (analytic cohort). Participants were recruited into CLUE I and II in 1974 and 1989, respectively, in Washington County, MD [34, 35]. These two cohorts were designed to study cancer, stroke, and heart disease using serum markers as precursors. CLUE I recruited 26 147 individuals to whom a brief health history questionnaire was administered and blood pressure was measured [46]. In CLUE II, a 20-ml heparinized blood specimen was collected from 32 894 individuals and stored at −70°C [47]. Of them, 8394 individuals participated in both studies. Compared to the 1980 Census and the 1990 Census, both CLUE I and II cohorts recruited ∼30% of adult residents in the county. This study was approved by the Institutional Review Board at the Johns Hopkins Bloomberg School of Public Health. Informed consent was obtained from each study participant.

### Lung cancer ascertainment and selection of cases and matched controls

Incident cancer cases were ascertained by linkage with the Washington County Cancer Registry and, since 1992, with the Maryland Cancer Registry. Cancer deaths were identified initially from state vital statistics, next of kin, and obituaries. Underlying cause of death was abstracted from death certificates. In the analytic cohort, 241 first primary lung cancer cases were ascertained through January 2018. Matched controls were sampled using incidence density sampling among participants in the analytic cohort who were alive and did not have lung cancer at the case’s date of lung cancer diagnosis. Controls were matched with cases on age (within 3 years), sex, smoking status and intensity, and date of blood draw (within 4 months). Of 241 case-control pairs, 208 cases and their matched controls had a sufficient DNA amount and quality for DNA methylation profiling and were, therefore, included in this analysis.

### DNA methylation measurement and quality control

We used peripheral blood leukocyte DNA methylation data previously measured by CLUE II using the Illumina 850K Infinium MethylationEPIC (EPIC) BeadChip Array. Four principal components (PCs) were generated with control for surrogate variable analysis to explain the batch effect variation in the data. These four PCs were used in the downstream analysis. Details of DNA methylation measurements, data preprocessing, and quality control procedures were provided previously [36].

### Calculation of the methylation scores for prenatal smoking exposure

To our knowledge, six sets of CpGs with blood DNA methylation patterns reflecting prenatal smoking exposure have been identified [26–31]. These methylation signatures were developed irrespective of health outcomes. Of these, we derived two methylation scores for use in our analysis. The first, called “Score-19,” included CpGs identified by the Pregnancy And Childhood Epigenetics consortia on the Illumina Infinium HumanMethylation450K BeadChip Array (HM450) measurement platform [26] to show differences between neonate cord blood and peripheral blood collected at 7 [26] and 30 years of age [30], on average, with prenatal smoking exposure. This was chosen because it was the best powered study for discovery of CpG methylation differences associated with prenatal exposure and because it reflects prenatal exposure effects only (neonates had no postnatal exposure). However, because we were interested in using methylation patterns present in adult peripheral blood (and the former work was discovered in neonate cord blood), we decided to also derive a score from CpGs identified in adult blood showing methylation-level associations with prenatal smoking exposure that occurred 30 years prior, while these adults were *in utero* [30]. This score, called “Score-15,” consisted of 15 CpG sites with an false discovery rate < 5% identified in the study by Richmond *et al*. [30]. For Score-15 and Score-19, 13 of the 15 and 18 of the 19 CpGs were available in the CLUE II clean methylation data profiled using the EPIC array. There were a total of eight CpGs overlapped in both Score-15 and Score-19, of which seven were available in CLUE II. Methylation smoking scores, Score-15 and Score-19, were calculated by taking the sum of the product of the effect size (reported in the studies by Richmond *et al*. and Joubert *et al*.[26, 30]) and the methylation beta values. A higher Score-15 or Score-19 indicates a higher exposure of prenatal smoking.

### Calculation of the methylation scores for adult active smoking history

We also calculated a methylation score to predict adult active smoking history (in packyears) to enable us to assess the independent effects of prenatal and personal smoking exposure history. We used a previously reported DNA methylation signature for smoking-related DNA methylation alterations [48], which was previously implemented in a CLUE II study [36, 49]. This methylation smoking score was also calculated by taking the sum of the product of the effect size and the methylation beta values, with a higher score indicating higher adult active smoking history.

### Statistical analysis

We divided the distribution of the cases’ year of birth (1905–61) into quartiles, forming four birth cohorts, from Quartile 1 (Q1; most recent) to Quartile 4 (Q4; oldest). Means or proportions were calculated for demographic characteristics, lung cancer–related risk factors, and the two prenatal smoking methylation scores for cases and controls overall and by birth cohort. Spearman correlations between the prenatal smoking scores and birth year were calculated separately for cases and controls. Conditional logistic regression was used to estimate OR of lung cancer. We calculated 95% CIs per 1-SD increase in the prenatal smoking scores, adjusting for body mass index (BMI), methylation-predicted active smoking history (in packyears), and the four PCs for the batch effect variation. We estimated the association for prenatal smoking scores overall and within each of the four birth cohort strata. Subgroup analyses were performed restricting to ever smokers (*N* = 186 pairs), restricting to cases with NSCLC and their matched controls (*N* = 155 pairs), and stratifying by quartiles of age at diagnosis. To rule out potential reverse association for the finding, we also conducted a sensitivity analysis excluding those pairs with the cases diagnosed within 5 years after the blood draw.

To increase the likelihood that a participant had or did not have prenatal smoking exposure, we jointly classified the participants using the two prenatal smoking methylation scores based on the median combined over cases and controls as the cut point (i.e. low–low [referent], low/high or high/low, and high–high). We used the median because no cut point that indicates prenatal exposure has been established. To determine the combined effects of active smoking and prenatal smoking on lung cancer risk, we derived dichotomous categories of prenatal and personal active smoking exposure, using DNA methylation scores, as follows: (i) low on both prenatal scores [referent] vs all other combinations, with cut points at the median, and (ii) low adult packyears methylation score [referent] vs high, with the cut point at the median or at the 67th percentile). The cut points were defined using both the cases and controls. Some of the CpGs in the prenatal smoking scores are in genes that are also linked with active smoking (*AHRR, MYO1G, CYP1A1*, *FRMD4A*, and *GFI1*) [39–41]. Thus, we assessed whether associations between the prenatal smoking scores and lung cancer risk are explained by those CpGs in genes related to active smoking. We restricted to ever smokers and evaluated the association of (i) Score-15 and Score-19 excluding CpGs in genes linked with active smoking and (ii) scores for the CpG in each active smoking-related gene one at a time with lung cancer risk.

Statistical analyses were conducted using SAS version 9.4 (RRID:SCR_008567) and R Project for Statistical Computing version 4.2.3 (RRID:SCR_001905). All hypothesis tests were two sided with a Type-I error rate of 0.05.

## Supplementary Material

dvae015_Supp

## Data Availability

The datasets generated during the current study are available from the corresponding author on reasonable request.
