# Supplementary material for: Prenatal exposure to maternal smoking and adult lung cancer risk: a nested case-control study using peripheral blood leukocyte DNA methylation prediction of exposure
Source: Environ Epigenet. 2024 Sep 20;10(1):dvae015. doi: 10.1093/eep/dvae015 (PMC11562842; doi:10.1093/eep/dvae015)
Supplement: dvae015_Supp [file dvae015_supp.zip › suppl_data/eap_Supplementary Tables revised_1.docx]

**Supplementary material**

Supplementary Table 1. Baseline characteristics of 208 pairs of lung cancer cases and matched controls nested in CLUE II, 1989.

Supplementary Table 2. Associations between prenatal smoking scores and lung cancer risk overall and by birth cohort, in the 186 matched pairs nested in the CLUE II cohort (1989-2018) with cases diagnosed after 5 years since blood draw.

Supplementary Table 3. Associations between joint categories of prenatal smoking scores and adult packyear score1 and lung cancer risk, among ever smokers, 186 matched pairs nested in the CLUE II cohort (1989-2018).

Supplementary Figure 1. DNA methylation-predicted scores of active smoking history (in packyears) by birth year in 208 matched pairs of lung cancer cases and controls, nested in CLUE II, 1989.

| **Supplementary Table 1. Baseline characteristics of 208 pairs of lung cancer cases and matched controls nested in CLUE II, 1989.** | | |
| --- | --- | --- |
| **Characteristic** | **Case**, N = 208^1^ | **Control**, N = 208^1^ |
| **Age** (Median (Q1, Q3)), years | 59 (51, 66) | 56 (48, 63) |
| **Sex** |  |  |
| Female | 54% | 54% |
| Male | 46% | 46% |
| **Race** |  |  |
| Black | 1% | 0% |
| White | 99% | 100% |
| **Smoking status** |  |  |
| Never | 11% | 11% |
| Former | 38% | 38% |
| Current | 51% | 51% |
| **Self-reported packyears (ever smokers)^2^** (Median (Q1, Q3)) | 34 (12-51) | 25 (6, 40) |
| **Methylation-predicted score for personal packyears of smoking**  (Median (Q1, Q3)) | 8 (-29, 42) | -8 (-41, 21) |
| **BMI** (Median (Q1, Q3), kg/m^2^) | 26 (23, 29) | 26 (23, 29) |
| **BMI Category** |  |  |
| Normal | 44% | 42% |
| Overweight | 42% | 40% |
| Obese | 14% | 18% |
| ^1^Cases and controls were matched on sex, age (+/- 3 years), smoking status and number of cigarettes smoked per day (for 2 pairs with missing number of cigarettes smoked per day, their methylation-predicted packyear score were used for matching instead)  ^2^Ever smokers are defined as being either a current or former smoker.  BMI= body mass index; Q1=quartile 1, Q3=quartile 3. | | |

| **Supplementary Table 2.** Associations between prenatal smoking scores and lung cancer risk overall and by birth cohort, in the 186 matched pairs nested in the CLUE II cohort (1989-2018) with cases diagnosed after 5 years since blood draw. | | | | | | | | | |
| --- | --- | --- | --- | --- | --- | --- | --- | --- | --- |
| **Score-15**^1^ | | | | | **Score-19**^1^ | | | | |
|  | **Pairs N** | **OR**^2^ | **95% CI**^2^ | **p** |  | **Pairs N** | **OR**^2^ | **95% CI**^2^ | **p** |
| Overall | 186 | **1.30** | **1.01, 1.67** | **0.04** | Overall | 186 | 1.17 | 0.91, 1.50 | 0.2 |
| *By Birth Cohort Quartile^3^* | | | | | | | | | |
| Q1: 1938-1961 | 51 | 1.16 | 0.57, 2.36 | 0.7 | Q1: 1938-1961 | 51 | 0.76 | 0.39, 1.48 | 0.4 |
| Q2: 1930-1938 | 48 | **3.78** | **1.54, 9.28** | **0.004** | Q2: 1930-1938 | 48 | **2.46** | **1.17, 5.17** | **0.02** |
| Q3: 1923-1930 | 45 | 0.81 | 0.41, 1.60 | 0.5 | Q3: 1923-1930 | 45 | 0.98 | 0.54, 1.77 | 0.9 |
| Q4: 1905-1923 | 42 | 0.92 | 0.54, 1.57 | 0.7 | Q4: 1905-1923 | 42 | 0.94 | 0.55, 1.63 | 0.8 |
| ^1^ Score-15 was generated from CpGs discovered in adult biospecimens that were associated with their prenatal smoking exposure status, and shown to be independent of own active personal smoking history or passive secondary sources of smoking exposure (Richmond, 2018). Score-19 uses CpGs that were discovered in neonate cord blood and shown to also be detectable into adulthood and independent of own personal active smoking history (Joubert, 2016).  ^2^ **OR**: OR per SD. **CI**: Confidence interval. Models were adjusted for BMI, adult packyears score and principal components for batch effects.  ^3^ Matched control was placed in the same quartile as the case. | | | | | | | | | |

| **Supplementary Table 3.** Associations between joint categories of prenatal smoking scores and adult packyear score^1^ and lung cancer risk, among ever smokers, 186 matched pairs nested in the CLUE II cohort (1989-2018). | | | | |
| --- | --- | --- | --- | --- |
|  | **Cases/controls** | **OR**^2^ | **95% CI**^2^ | **p-value** |
| Low, referent: Prenatal: both prenatal smoking scores below the median, Adult: median | | | | |
| Low Prenatal, Low Personal active^1^ | 25/53 | Reference | — |  |
| Low Prenatal, High Personal active^1^ | 36/30 | 1.84 | 0.69, 4.93 | 0.2 |
| High Prenatal, Low Personal active^1^ | 56/52 | 2.81 | 1.38, 5.72 | 0.004 |
| High Prenatal, Low Personal active^1^ | 69/51 | 2.67 | 1.03, 6.95 | 0.04 |
| Low, referent: Prenatal: both prenatal smoking scores below the median, Adult: bottom 33% | | | | |
| Low Prenatal, Low Personal active^1^ | 17/37 | Reference | — |  |
| Low Prenatal, High Personal active^1^ | 44/46 | 1.09 | 0.41, 2.90 | 0.9 |
| High Prenatal, Low Personal active^1^ | 35/34 | 2.70 | 1.17, 6.23 | 0.020 |
| High Prenatal, Low Personal active^1^ | 90/69 | 1.99 | 0.79, 5.02 | 0.2 |
| ^1^: Dichotomous categories of smoking exposure using DNA methylation scores were created to reflecte both prenatal and personal exposure to smoking as follows: (i) low on both prenatal scores [referent] vs all other combinations, with cutpoints at the median), (ii) low personal smoking packyears [referent] vs high, with the cutpoint at the median or at the 67^th^ percentile (the cutpoints were defined using both the cases and controls). Two prenatal smoking methylation scores were Score-15, initially identified in adult biospecimens, and Score-19, initially identified in neonate/child blood specimens and confirmed to persist into adulthood.  ^2^: **OR**: OR per SD. **CI**: Confidence interval. Models were adjusted for BMI, adult packyears score, and principal components. | | | | |

**Supplementary Figure 1. DNA methylation-predicted scores of active smoking history (in packyears) by birth year in 208 matched pairs of lung cancer cases and controls, nested in CLUE II, 1989.**

**
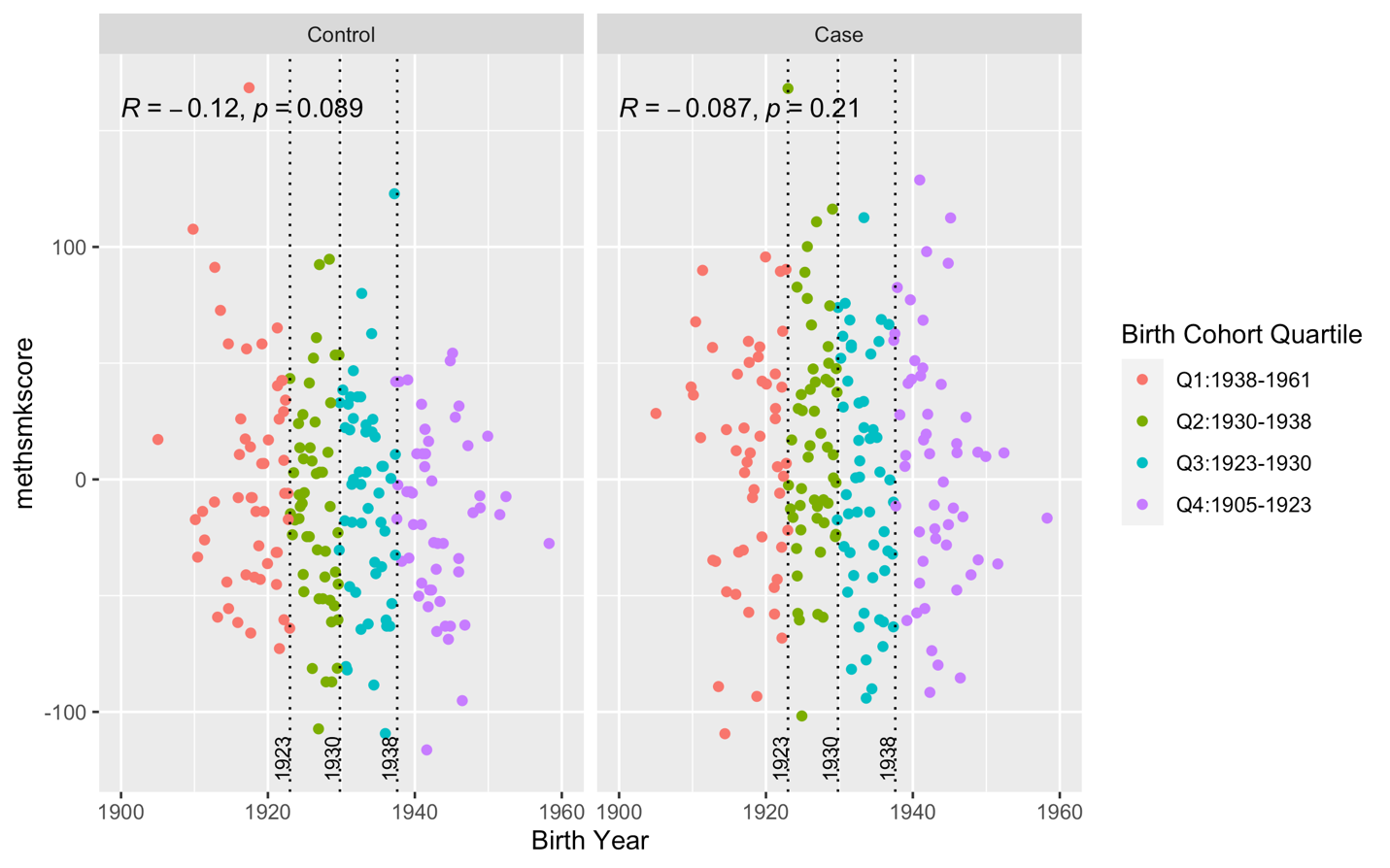
**
